# Estrogen Receptor and Vascular Aging

**DOI:** 10.3389/fragi.2021.727380

**Published:** 2021-09-24

**Authors:** Morgane Davezac, Melissa Buscato, Rana Zahreddine, Patrick Lacolley, Daniel Henrion, Francoise Lenfant, Jean-Francois Arnal, Coralie Fontaine

**Affiliations:** ^1^ INSERM-UPS UMR U1297, Institut des Maladies Métaboliques et Cardiovasculaires, Université de Toulouse, Toulouse, France; ^2^ INSERM, UMR_S 1116, DCAC Institute, Université de Lorraine, Vandœuvre-lès-Nancy, France; ^3^ INSERM U1083 CNRS UMR 6015, CHU, MITOVASC Institute and CARFI Facility, Université d’Angers, Angers, France

**Keywords:** estradiol, estrogen receptor, menopause, atherosclerosis, endothelium, vascular aging

## Abstract

Cardiovascular diseases remain an age-related pathology in both men and women. These pathologies are 3-fold more frequent in men than in women before menopause, although this difference progressively decreases after menopause. The vasculoprotective role of estrogens are well established before menopause, but the consequences of their abrupt decline on the cardiovascular risk at menopause remain debated. In this review, we will attempt to summarize the main clinical and experimental studies reporting the protective effects of estrogens against cardiovascular diseases, with a particular focus on atherosclerosis, and the impact of aging and estrogen deprivation on their endothelial actions. The arterial actions of estrogens, but also part of that of androgens through their aromatization into estrogens, are mediated by the estrogen receptor (ER)α and ERβ. ERs belong to the nuclear receptor family and act by transcriptional regulation in the nucleus, but also exert non-genomic/extranuclear actions. Beside the decline of estrogens at menopause, abnormalities in the expression and/or function of ERs in the tissues, and particularly in arteries, could contribute to the failure of classic estrogens to protect arteries during aging. Finally, we will discuss how recent insights in the mechanisms of action of ERα could contribute to optimize the hormonal treatment of the menopause.

## Introduction

**FIGURE 1:**
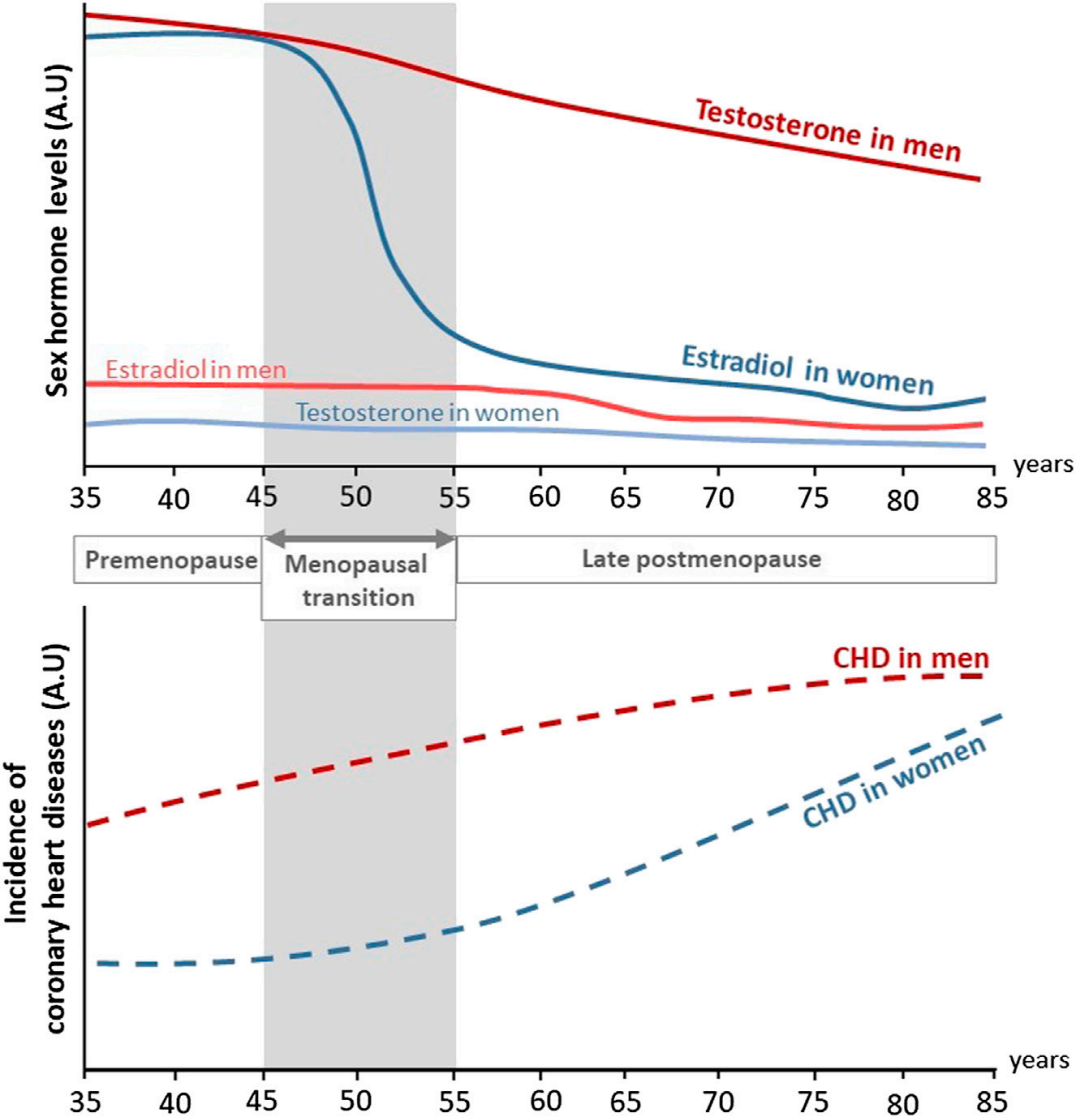
Evolution of sex hormones levels and incidence of coronary heart diseases in men and women through life. During their life, women are at lower risk to develop coronary heart diseases (CHD) compared to men. In both sexes, aging is associated to an increased incidence of CHD. In addition, while testosterone levels decrease progressively with aging in men, estradiol levels rapidly drop at the onset of menopause in women and is associated to a rise in CHD (adapted from Lerner and Kannel (1986)). A.U, Arbitrary unit.

Aging plays a critical role in the deterioration of arteries function, resulting in an increased risk of cardiovascular diseases (CVD) in both men and women. Mechanisms of vascular aging have been extensively described and are mainly characterized by endothelial dysfunction, *i.e.* loss of vasorelaxation, and large artery stiffening (Lacolley et al., 2017; Donato et al., 2018). Sex differences also play a critical role in both onset and prevalence of CVD (Lerner and Kannel, 1986; Virani et al., 2021). Women are protected against CVD before the onset of menopause. Overall, differences in CVD risk factors and outcomes between men and women are largely attributed to sex hormones and their associated receptors. In particular, this protection has been largely attributed to estrogens, which exert many beneficial effects on the arterial wall including vasodilation, atheroprotection and acceleration of endothelial healing in response to injury which will be developed in this review. Accordingly, postmenopausal deprivation of estrogens may be related to the increased CVD risk in aging women (Newson, 2018). Indeed, serum levels of 17β-estradiol (E2), the main circulating estrogen, drop rapidly at the onset of menopause in women. Because of this abrupt decline, women loss their vascular benefits compared to men and incidence of CVD in postmenopausal women is 4.3 times higher than that in premenopausal women (Lerner and Kannel, 1986) (Figure 1). Accordingly, elderly women are reported to be at similar risk for CVD than age-matched men: in adults above 80 years of age, the incidence of CVD was reported to be 89.4% in males, and 90.8% in females with hypertension in 2018 in US (Virani et al., 2021). One of the mechanism involved is the attenuation of pulse pressure amplification due to increased arterial stiffness in postmenopausal women (Regnault et al., 2012). XX chromosome was recently attributed to increased atherosclerosis in mice (AlSiraj et al., 2019), which could also exacerbate the detrimental effect of estrogen deprivation on the cardiovascular system.

Effects of estrogens are mediated by the Estrogen Receptors alpha (ERα) and beta (ERβ). Both ERs are expressed in the endothelium and smooth muscle cells of human coronary arteries (Losordo et al., 1994; Kim-Schulze et al., 1996), however ERα has been shown to mediate most of the vascular protective effects of E2 (Arnal et al., 2010). ERα belongs to the nuclear receptor superfamily and acts classically as a transcription factor, but it can also exerts extranuclear, non-genomic actions by activating rapid membrane-initiated steroid signaling (MISS), as demonstrated specifically in the endothelium (Dan et al., 2003; Arnal et al., 2017). Beside the decline of estrogens, abnormalities in the expression and/or signaling of ERs due to aging could contribute to the failure of classic estrogens to protect arteries. Although some studies show a decrease of ERα in immune and endothelial compartments with age (Gavin et al., 2009; Bowling et al., 2014), these changes remain poorly described in both women and animal models. ERα also plays a role in arterial protection in males through aromatisation of testosterone into E2 (Mendelsohn and Karas, 2005). Testosterone levels decrease gradually during life in men (1% every year starting at 40), however consequences of testosterone below the normal range in elderly remain debated due to great variability between individuals and age-related confounding factors (Kaufman and Vermeulen, 2005; Perheentupa and Huhtaniemi, 2007).

Thus, understanding the interplay between estrogen deprivation, ERα regulation and aging of the cardiovascular system is essential for the development of new therapeutic strategies. Two phenotypes hallmark aging, atherosclerosis, which is a local development of plaques, and arteriosclerosis which develops all along the arterial tree. They share similar changes in hemodynamic factors, smooth muscle cells plasticity and calcification. A particular trait of atherosclerosis is the involvement of multiple cell types, endothelial and smooth muscle cells, fibroblasts but also extravascular cells. For arteriosclerosis, it appears simpler since it is primarily mediated by structural changes of the media which is a privileged site, avascular, with only smooth muscle cells and without leucocytes. The onset of these two phenotypes occurs in parallel, even though, several reports indicate that arterial stiffness precedes age-related full plaque development. In this review, we will summarize the main clinical and experimental studies reporting the protective effects of sex hormones on the arterial wall focusing mainly on endothelial cells, the main cellular target of estrogens. Accordingly, the impact of aging on endothelial healing and prevention of atherosclerosis by estrogens will be more particularly described. Finally, we will discuss how recent insights in the mechanisms of action of ERα could contribute to optimize the hormonal treatment of menopause.

## Clinical Evidence of Vascular Protection Conferred by Female Hormones in Women

### Clinical Evidence of Female Hormones Protection Against Cardiovascular Diseases

The Framingham Heart Study was initiated in 1948 and aimed at elucidating epidemiology and risk factors of CVD (Mahmood et al., 2014). Sex differences in CVD risks, incidence and mortality were assessed on the initial cohort of 5127 participants followed over a period of 26 years (Lerner and Kannel, 1986). During this period, the incidence of CVD in men was twice the one in women. Clinical manifestation of CVD between sexes were also different: the dominant cardiovascular event in men was acute myocardial infarction, while stable angina pectoris was preponderant in women. Strikingly, after 45–55 years old the difference in CVD risk between sexes narrowed and this was associated to a rise of CVD incidence in women during the decade following menopause. However, the direct impact of menopause transition on CVD risk was not assessed in this study. The Framingham Heart Study also identified smoking, hyperlipidemia, hypertension and diabetes as major risk factors for developing CVD (Lerner and Kannel, 1986), which ones are also submitted to sex differences. For example, even if total cholesterol is superior in women than in men, women present a more favorable lipid profile with higher levels of HDL-cholesterol and lower levels of LDL-cholesterol (Virani et al., 2021). Regarding hypertension (defined by systolic blood pressure ≥140 mm Hg or diastolic blood pressure ≥90 mm Hg), the prevalence in men is twice the one of women aged 20–34 and become superior in women only after the age of 65 (Virani et al., 2021). However, the lower prevalence of risk factors in women cannot explain alone their protection against CVD. For example, the ELSA-Brazil study (Brazilian Longitudinal Study of Adult Health) examined a population of low-risk individuals lacking common CVD risk factors and found that men still have higher carotid intima-media thickness (IMT), a marker of atherosclerosis, than women (Santos et al., 2014). Besides, recent epidemiological studies continue to show that young women are protected against CVD compared to their male counterparts. Until the age of 59, prevalence of CVD (including coronary heart disease, heart failure and stroke) is systematically lower in women, but after 60 years old, prevalence in women catch-up with men (Virani et al., 2021). Altogether, these different studies revealed sex difference in the incidence of CVD but this difference tends to disappear with menopause (Figure 1 and Table 1).

**TABLE 1:** Clinical evidence of cardiovascular protection conferred by female hormones. CVD, cardiovascular diseases; IMT, intima-media thickness; CAC, coronary artery calcification; FMD, flow-mediated dilation.

	Study Name	Results	References
Global CVD risk	Framingham Heart Study	Incidence of CVD in men is twice the one in women until 45–55 years	Lerner and Kannel, (1986)
	Heart Disease and Stroke Statistics—2021 update.	Prevalence of CVD is lower in women before 60 years old. Women have lower incidence of CVD risk factors such as high LDL cholesterol and hypertension	Virani et al. (2021)
	Elsa-Brazil Study	Among low-risk individuals, young women still present lower carotid IMT than men	Santos et al. (2014)
	Nurses′ Health Study	Bilateral oophorectomy increases the risk of CVD	Belanger et al. (1978)
Subclinical atherosclerosis	Framingham Heart Study	Prevalence of CAC is lower in women compared to men	Hoffmann et al. (2008)
	Bogalusa Heart Study	Lower IMT in young women compared to men	Urbina et al. (2002)
	InterLACE consortium	Higher risk of coronary heart diseases in women with premature menopause	Zhu et al. (2019)
	Cross-sectional study	No difference in IMT between men and postmenopausal women	Stamatelopoulos et al. (2012)
Endothelial function	Cross-sectional study	Decline in FMD occurs latter in women than in men	Celermajer et al. (1994)
	Cross-sectional study	In early postmenopausal women (<3 years in menopause), FMD was decreased by 40% compared to age-matched premenopausal women	Bechlioulis et al. (2010)

Menopause is marked by the permanent end of the menstrual cycles in women and occurs generally around the age of 49–52. Following the loss of ovarian follicles, ovaries cease to produce estrogens and the circulating levels of E2, that normally vary between 60 and 200 pg/ml across the menstrual cycle, rapidly drop to 20 pg/ml (Randolph et al., 2011). However, studying the direct cardiovascular consequences of menopause in clinical trials is complicated because difference of age between premenopausal and postmenopausal women is a confounding factor, making hard to uncouple the effect of aging and of estrogen deprivation by itself on the vascular function. Moreover, transition to menopause takes several years and consists of 4 stages: early perimenopause, late perimenopause, early postmenopause and late postmenopause. According to the Stages of Reproductive Aging Workshop (STRAW) classification, early and late perimenopause correspond to the years around menopause and are characterized by menstrual cycles and endocrine changes. Early postmenopause corresponds to the 5 years after menopause. Late postmenopause is the final stage at which hormonal levels are stabilized (Harlow et al., 2012). In the Nurses’ Health Study, 121,700 women aged 30–55 were followed during 6 years in order to directly evaluate the impact of estrogen deprivation on CVD risk (Belanger et al., 1978; Colditz et al., 2010). Authors reported that surgical menopause induced by bilateral oophorectomy leads to an increased CVD risk compared to premenopausal women of the same age, but women with natural menopause have no such elevated risk. However, this cohort was relatively young, and authors correctly highlighted the need to study the effect of the duration of menopause.

### Clinical Evidence of Female Hormones Protection Against Atherosclerosis

The leading cause of the majority of CVD is atherosclerosis. Atherosclerosis is characterized by the accumulation of lipids in the arterial wall but is also a chronic inflammatory disease that leads to the formation of vessel-occluding plaques within the sub-intimal space of middle-sized and larger arteries. This space is normally devoid of any cell population. Two markers are classically used to evaluate atherosclerosis extent in patients: 1) increase in carotid IMT, considered as an early marker of subclinical atherosclerosis and 2) coronary artery calcification (CAC) which evaluates the presence of calcified lesions in the coronary arteries (Table 1). Among subjects from the Framingham Heart Study Offspring and Third Generation cohorts, prevalence of CAC was of 32.0% in women and 52.9% in men aged 35–70 years old. Moreover, severity of CAC was systematically lower in women in all age groups (Hoffmann et al., 2008). In the Bogalusa Heart Study, carotid IMT was measured in a cohort of young men and women of different ethnicities. Results show that male sex was systematically associated with higher IMT in all segments of the carotid artery (Urbina et al., 2002). Zhu and colleagues analysed the occurrence of coronary heart diseases among postmenopausal women from 15 observational studies. They found that women who had premature menopause (age <40 years) or early menopause (age 40–44 years) were at higher risk of developing coronary heart diseases compared to women who had menopause at age 50–51 (adjusted hazard ratios of 1.52 and 1.30 respectively) (Zhu et al., 2019). In another study by Stamatelopoulos and colleagues, comparison of postmenopausal women (<10 years after menopause) and age-matched men showed no difference in carotid IMT and the number of plaques, highlighting that the atheroprotection conferred to premenopausal women is rapidly lost after menopausal transition (Stamatelopoulos et al., 2012).

### Clinical Evidence of Female Hormone Protection on Endothelial Function

Endothelium is the guardian of vascular integrity by regulating the vascular tone but also through its anti-inflammatory and anti-thrombotic properties. Endothelium dysfunction is mainly characterized by the loss of endothelium-dependent vasorelaxation and is considered as a predictor marker of numerous cardiovascular diseases, including atherosclerosis (Schächinger et al., 2000). Endothelium function, assessed by brachial flow-mediated dilation (FMD), has been shown to decrease with age. While this decline occurs as soon as 40 years old in men, FMD in women is stable until 50 years old but decreases faster at the time of menopause compared to men (0.49%/year in women vs 0.21%/year in men) (Celermajer et al., 1994). Interestingly, in a study of young women in which FMD was measured at different times of the menstrual cycle, increased endothelium-dependent vasodilation was observed in the follicular and luteal phases of the menstrual cycle, where serum estradiol levels are high (Hashimoto et al., 1995). In early postmenopausal women (<3 years in menopause), FMD was decreased by 40% compared to age-matched premenopausal women, indicating that endothelial function is impaired rapidly after estrogen deprivation (Bechlioulis et al., 2010). Interestingly, no difference in carotid IMT was found in this cohort, suggesting that endothelial dysfunction appears prior to structural atherosclerosis changes (Bechlioulis et al., 2010). This was confirmed in another study, in which a decrease in FMD was already observed in late perimenopausal women and was further worsened in early and late postmenopausal women (Moreau et al., 2012).

Overall, considerable epidemiological evidence has accumulated regarding the protective role of female sex hormones against cardiovascular diseases at least in part by the protection they exert on the endothelium and against atherosclerosis (Table 1). To which extent estrogen deprivation due to menopause contributes to arterial aging and increase of cardiovascular disease remains to be clearly established. Indeed, as the menopausal transition occurs over several years, it is challenging to separate the effects of biological aging from the hormonal status.

## From Observational Studies on Hormonal Replacement Therapy to the Timing Hypothesis of the Women’s Health Initiative

The direct effect of female sexual hormones has been evaluated studying the impact of hormonal treatment in menopausal women. Indeed, menopause symptoms can be alleviated or even totally suppressed by menopausal hormone therapy (MHT), initially based on natural estrogens extracted from the urine of pregnant mares (mainly in the United States, using the oral route) and later from the synthesis of the natural estrogen, E2 (mainly in Europe, in particular using the transdermal route). Several observational studies first suggested that MHT, based on the administration of estrogens and progestin, could prevent the rise of CVD in postmenopausal women (reviewed in Hale and Shufelt (2015)). In one of the largest cohort, the Nurses' Health Study (NHS) (enrolling during 1980–1994 and following participants until 2002), women on MHT had a 45% reduction in CVD risk (Grodstein et al., 2000). However, the major questions arising from this cohort data were whether MHT protects older women or women with established CVD risks. Other smaller cohorts, as the Cardiovascular Health Study (CHS) and the Rotterdam Study, have also associated the use of MHT to favorable CVD risk factors including lower LDL-cholesterol, higher HDL-cholesterol and decreased carotid IMT (Manolio et al., 1993; Westendorp et al., 1999).

Since observational studies have pointed out that MHT may play a role in primary CVD prevention and due to increased number of prescriptions of these treatments, the National Institutes of Health started the Women’s Health Initiative (WHI) study to examine the potential risks and benefits associated with such menopausal treatment in 2002. The study was divided in two arms: in the first arm, 16,000 menopausal women aged 50–79 years were randomized to conjugated equine estrogens (CEE) with medroxyprogesterone acetate (MPA) *versus* placebo, and in the second arm, women who had hysterectomy were randomized to CEE alone *versus* placebo. First part of the study (CEE+MPA) was stopped early in 2002, because of an increase in the incidence of thromboembolism, breast cancer and, quite unexpectedly, CVD (Rossouw et al., 2002). Seven years after starting the second arm of the study (CEE alone), no increase in CVD was found, demonstrating an important role for MPA in the rise of CVD (Anderson et al., 2004). Reanalysis of the clinical trial highlighted the importance of treatment initiation timing. Indeed, estrogens may have a beneficial effect on the heart if started at early menopause, when arteries are relatively healthy, but a harmful effect if started at late menopause, when those arteries are more likely to show signs of atherosclerotic disease (Rossouw et al., 2007). Some interventional studies went in the same direction, showing a decrease in FMD in response to acute and chronic E2 in women menopaused for more than 5 years (Sherwood et al., 2007; Vitale et al., 2008). Finally, the “timing hypothesis” was confirmed in 2016 by the Early *versus* Late Intervention Trial with Estradiol (ELITE) group results, showing beneficial effects of E2 therapy on carotid atherosclerosis in early (≤6 years) compared to late (≥10 years) menopausal women (Hodis et al., 2016). Recent evidence has also highlighted the role of chronological aging *per se* on CVD risk factors independently of hormonal status. By comparing not only pre-*versus* postmenopausal women, but also women of same menopausal status and of different age, de Kat and coworkers proposed that menopause and age are independently associated to an increase in CVD risk factors such as high cholesterol, high blood pressure and obesity (de Kat et al., 2017).

Altogether, observational and interventional studies clearly established that MHT has beneficial impact on CVD prevention if initiated early after the onset of menopause. Unanswered questions remain to be determined regarding the differential effect of hormones during aging of the arterial wall, thereby requesting further studies using experimental models.

## Vasculoprotective Effects of Estrogens in Experimental Models

Except for humans, menopause has been observed only in four other species in the wild: beluga whales, narwhals, orcas and short-finned pilot whales (Ellis et al., 2018a; 2018b). The evolutionary advantage that could represent menopause has been subject to intensive debate. Among theories, the “Grandmother Hypothesis” was put forward by evolutionary biologists arguing that women who lost the capacity to reproduce can help to take care of their grandchildren, enhancing their survival rate and by doing so, increasing their genetic and epigenetic contribution to the next generations (Lahdenperä et al., 2004). Menopause, clinically interpreted as the permanent cessation of menstruation in age-appropriate women, is caused by a decline in ovarian follicular activity. Ovaries produce and release two kinds of sex hormones—progesterone and estrogens. The naturally occurring estrogens in women are estrone (E1), E2, estriol (E3) and estetrol (E4). E2, synthesized primarily in the ovaries, is the most abundant estrogen in nonpregnant females of reproductive age. Upon cessation of ovarian function at menopause or following surgical ovariectomy, estrone (E1) is produced in peripheral tissues from the adrenal androgen and represents the principal circulating estrogen. During pregnancy, estriol (E3) is produced by the placenta and estetrol (E4) by the fetal liver, but production of this latter estrogen is restricted to the great ape. The use of ovariectomy, removal of both ovaries, as a model of menopause is widespread throughout the preclinical field for evaluating sexual hormone effects in female animal models. However, it is important to note that most studies are conducted in young ovariectomized females and therefore do not take into account the aging process associated to menopause, thus constituting a major limitation of this model.

### Impact of Duration of Estrogen Deprivation and Aging on Beneficial Vascular Action of E2

Endothelial-dependent vasodilation depends on the release of the vasodilator mediator nitric oxide (NO), produced by the endothelial Nitric Oxide Synthase (eNOS). Sex difference in basal NO release was demonstrated in rabbit aortic rings (Hayashi et al., 1992) and perfused rat aortas (Kauser and Rubanyi, 1994), showing that female arteries produced more NO than male arteries. In addition, the use of ovariectomy abrogated this sex difference, leading to a decreased relaxation compared to intact females (Hayashi et al., 1992). The direct demonstration that E2 exerts vasodilatory effects came from Gisclard and colleagues, who showed that E2 enhances endothelium-dependent dilation of rabbit femoral arteries *ex vivo* (Gisclard et al., 1988) (Table 2). Duration of estrogen deprivation impairs the response to E2 in healthy arteries, independently of any vascular structural alteration. For instance, in rats ovariectomized for 8 months, E2 treatment no longer improved endothelium-dependent relaxation in the aorta and this was not correlated to any endothelial damage (Pinna et al., 2008). In addition to estrogen deprivation, chronological aging of the arteries by itself could be responsible for the loss of E2 vascular response. Notably, different groups demonstrated that E2 vasodilatory effect is diminished in aged animal models. First, endothelial-dependent relaxation of the aorta and mesenteric artery were diminished by 20% in 18-month-old female intact mice compared to young female mice, suggesting that age impacts the response to endogenous estrogens (Guivarc’h et al., 2020). Second, relaxation response to exogenous E2 was decreased in aging female spontaneously hypertensive rats (SHRs) and was associated to decreased NO production (Wynne et al., 2004). Third, the vasodilatory effect of exogenous E2 was also decreased in 29-month-old mice uterine arteries and correlated with a basal decrease in endothelial-dependent relaxation and increased arterial stiffness, two features of vascular aging (Nicholson et al., 2017).

**TABLE 2:** Experimental evidence of E2 protection on arteries. OVX, ovariectomized; E2, 17β-estradiol.

Beneficial effect	Results	References
Nitric oxide release	Female > Male (rabbit)	Hayashi et al. (1992)
	Female > Male (rat)	Kauser and Rubanyi (1994)
	Female > OVX Female (rabbit)	Hayashi et al. (1992)
	OVX + E2 Female > OVX Female (rabbit)	Gisclard et al. (1988)
Endothelial healing	OVX + E2 Female > OVX Female (rat)	Krasinski et al. (1997)
	OVX + E2 Female > OVX Female (mouse)	Brouchet et al. (2001)
Flow-mediated outward remodeling	OVX + E2 Female > OVX Female (rat)	Tarhouni et al. (2014)
Prevention of atherosclerosis	Female > OVX Female (monkey)	Adams et al. (1985)
	OVX + E2 Female > OVX Female (monkey)	Williams et al. (1990)
	OVX + E2 Female > OVX Female (rabbit)	Fischer and Swain (1985), Haarbo et al. (1991)
	OVX + E2 Female > OVX Female (ApoE^−/−^ mice)	Bourassa et al. (1996), Elhage et al. (1997)
	OVX + E2 Female > OVX Female (LDLr^−/−^ mice)	Marsh et al. (1999), Billon-Galés et al. (2009a), Billon-Galés et al. (2009b)

Loss of vascular beneficial action of E2 after a prolonged period of estrogen deprivation was also demonstrated in flow-mediated outward remodeling (FMR) of resistance arteries in rats. In this experimental model, ovariectomy of 3-, 9-, and 12-month-old rats abolished FMR. This vascular action was restored, independently of age, by immediate E2 replacement but not if E2 was given 9 months after ovariectomy (Tarhouni et al., 2014). Thus, in this model, E2 deprivation, rather than age, leads to decline in FMR.

Overall, duration of estrogen deprivation and vascular aging are two parameters that can alter differently the estrogenic response of healthy arteries (Table 3). These results also suggest that estrogenic signaling could be altered after both prolonged estrogenic deprivation and aging.

**TABLE 3:** Impact of estrogen deprivation and aging on beneficial action of E2.

Beneficial action of estrogens on	Alteration by	References
Nitric oxide release	Duration of estrogen deprivation	Pinna et al. (2008)
	Chronological aging	Guivarc’h et al. (2020)
Flow-mediated outward Remodelling	Duration of estrogen deprivation	Tarhouni et al. (2014)
Endothelial Healing	Remains to be defined	

### Impact of Estrogens on Endothelial Healing in Animal Models

Endothelial barrier integrity is required for maintaining vascular homeostasis and fluid balance between the circulation and surrounding tissues. In contrast, abnormalities of endothelial function and loss of the integrity of the endothelial monolayer constitute the key step in the onset of atherosclerosis. Endothelial erosion is directly responsible for thrombus formation and cardiovascular events in about one-third of the cases of acute coronary syndromes. Thus, after endothelial injury, the vascular repair process, also called endothelial healing, is crucial to the restoration of endothelial junctions in order to reform semipermeable barrier and thus to prevent the development of vascular disease such as atherosclerosis or restenosis. The accelerative effect of E2 on endothelial healing was first demonstrated in ovariectomized rats, following balloon denudation of the carotid artery (Krasinski et al., 1997). These results were reproduced in female mice, in which E2 accelerated endothelial healing after electric injury of the carotid artery (Brouchet et al., 2001) (Table 2). In E2-treated female mice, this accelerative effect was associated to the retrograde commitment of an uninjured endothelial zone which rapidly migrates and proliferates (Filipe et al., 2008). Interestingly, eNOS was necessary for the acceleration of endothelial healing in response to E2 (Iwakura et al., 2003), but this effect was independent of eNOS enzymatic activity since eNOS inhibition by L-N^G^-nitro arginine methyl ester (L-NAME) failed to prevent acceleration of endothelial healing in response to E2, suggesting rather a role of scaffold protein for eNOS in this process (Billon et al., 2008). E2 also promoted recruitment of bone marrow-derived endothelial progenitor cells (EPCs) and their incorporation at the site of injury (Iwakura et al., 2003), however this finding was not confirmed by subsequent studies (Filipe et al., 2008). Overall, acceleration of endothelial healing in response to E2 probably relies on the interaction of local endothelial cells and circulating cells and the full mechanism has not been elucidated yet.

The deleterious effect of aging on the endothelial healing process was demonstrated for the first time in the rabbit iliac artery. Indeed, 28 days after balloon denudation of the iliac artery, reendothelialization was reduced by 40% in old (4-year-old) compared to young (7-month-old) male rabbits (Gennaro et al., 2003). Recently, McDonald and coworkers developed a new model for the study of endothelial regeneration in the abdominal aorta (McDonald et al., 2018). Combining this injury model with large scale transcriptomic approaches, they demonstrated that in young (8-week-old) mice expression of the transcription factor *Atf3* was necessary to achieve endothelial repair, while in aged (18-month-old) mice, decreased *Atf3* expression was associated to the retardation of endothelial regeneration. Nevertheless, how aging and/or duration of estrogen deprivation could affect endothelial healing in response to E2 remains to be defined.

### Impact of Estrogens on Prevention and Established Atherosclerosis in Animal Models

The consequence of ovariectomy on atherosclerosis was initially highlighted in 1985 in the monkey model. In adult female cynomolgus monkeys fed an atherogenic diet for 30 months, ovariectomy led to an increase in atherosclerotic lesions size in coronary, carotid and femoral arteries (Adams et al., 1985). Compared to female intact monkeys, lesions were 2–10 times greater in ovariectomized animals and led to the loss of “female protection” (Adams et al., 1985). Authors suggested that this protection was mediated by steroid hormones. The protective effect of E2 on atherosclerosis was demonstrated several years later by the decrease of atherosclerotic plaque extent thanks to supplementation of ovariectomized female monkeys with E2 (Williams et al., 1990). In addition to monkeys, rabbits were extensively used to study atherosclerosis as they develop rapidly atheromatous plaques in response to hypercholesterolemic diet. Studies in rabbits found similar results: cholesterol-fed female rabbits treated with E2 had a 60% reduction of aortic accumulation of cholesterol compared to the control group (Fischer and Swain, 1985; Haarbo et al., 1991). Treatment with a progestin had no effect on lesion size, further supporting that female cardiovascular protection is attributed to estrogen and not to progesterone (Fischer and Swain, 1985; Haarbo et al., 1991). With emergence of knock-out/in technologies, mice have then emerged as favorite model to study atherosclerosis. Two genetically modified models developing rapidly atherosclerotic lesions are largely used: apolipoprotein E–deficient (ApoE^−/−^) and low-density lipoprotein receptor-deficient (LDLr^−/−^) mice fed with a normal chow and cholesterol-enriched diet respectively. In ApoE^−/−^ female mice, chronic administration of E2 led to a reduction of average 75% of lipid deposition both at the aortic sinus and after *en face* analysis of the whole aorta (Bourassa et al., 1996; Elhage. et al., 1997). In LDLr^−/−^ female mice, E2 supplementation also reduced atherosclerotic plaque formation independently of plasma cholesterol levels (Marsh et al., 1999; Billon-Galés et al., 2009a) (Table 2). Results obtained from atherosclerotic coronary arteries from female monkeys treated with E2, initially lead to the hypothesis that NO mediates the atheroprotective effects of E2 (Williams et al., 1990). This hypothesis was finally rejected since E2 still conferred atheroprotection in double-deficient eNOS^−/−^ ApoE^−/−^ female mice (Hodgin et al., 2002), as well as in ApoE^−/−^ female mice treated with the NO inhibitor L-NAME (Elhage et al., 1997). In addition to lipid deposition, oxidative stress and inflammation also greatly contribute to the formation and progression of atherosclerosis. While the direct antioxidant effect of high concentrations of E2 (*i.e.* µM) is well established, physiological concentrations of E2 (nM range) elicit a prevention of oxidative stress in the endothelium, leading to an increase in NO bioavailability (Arnal et al., 1996). In addition, studies have produced conflicting results regarding its effect on inflammation. These two last points will not be developed here and have been reviewed elsewhere (Straub, 2007; Xiang et al., 2021).

Altogether, experimental models of atheroma with hypercholesterolemic monkeys, rabbits and mice demonstrated that E2 prevents atherosclerosis through a direct action on the arterial wall rather than through an effect on the lipid profile. However, these studies, realized in young adult animals, did not evaluate if aging or estrogen deprivation could interfere with E2 action. Only a few experimental studies have directly examined the impact of the timing of estrogen supplementation on the vasculature. Monkey studies performed by Clarkson’s group provided clear evidence that estrogen is effective in slowing the progression of coronary artery atherosclerosis only when administered soon after surgical menopause and that this benefit is lost if estrogen therapy is delayed. Indeed, in 26-month-old monkeys fed a hypercholesterolemic diet, CEE treatment led to a 70% atherosclerosis plaque reduction if started at the onset of ovariectomy but had no effect if delayed for 2 years, a time comparable to six postmenopausal years for women (Mikkola and Clarkson, 2002; Clarkson et al., 2013). Similar conclusions were obtained in 20-week-old ApoE^−/−^ mice in which E2 atheroprotective effect was lost when started after 45 days of estrogen deprivation (Cann et al., 2008), thus supporting the importance of timing of E2 initiation. Importantly, the work of Clarkson’s group demonstrated that the atheroprotective effect of estrogens is not conditioned by age but rather by the stage of progression of subclinical atherosclerosis, meaning that E2 can prevent the initiation of atherosclerosis in healthy arteries but is inefficient if the disease is already established. This demonstration was conducted in female monkeys in which atherosclerosis extent was measured before CEE treatment in the iliac artery, allowing to study the effectiveness of hormone treatment on the different stage of atherosclerosis (Clarkson et al., 2013). By contrast, studies in rabbits initially proposed that E2 exerts significant anti-atherogenic effect on established plaques measuring aortic total cholesterol content chemically (Haarbo and Christiansen, 1996). However, another study reported that, in rabbits ovariectomized and fed a cholesterol diet 84 days prior to E2 treatment, E2 was still able to reduce plaque formation in the carotid artery and thoracic aorta in which none or moderate plaques were established at the initiation of treatment, whereas E2 had no effect in the aortic arch in which severe atherosclerotic plaques had already developed (Hanke et al., 1999). Moreover, by contrast to healthy arteries (Hayashi et al., 1992), E2 was not able to induce endothelium-dependent dilation in rabbit arteries with atherosclerotic plaques (Holm et al., 1999), further supporting the conclusions obtained in the monkey model by Clarkson’s group.

**FIGURE 2:**
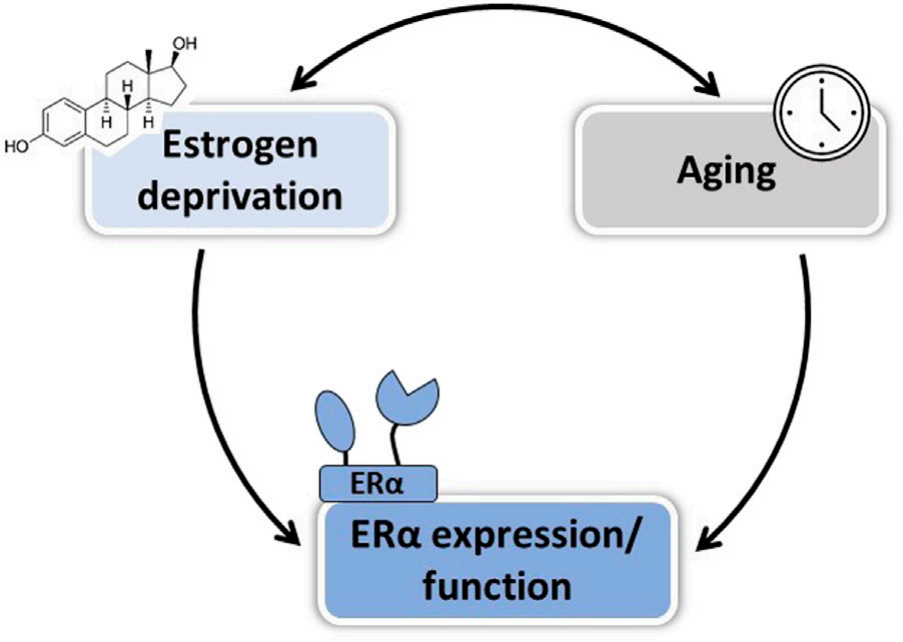
Factors involved in the loss of E2 vascular protection after menopause. Both duration of estrogen deprivation and aging *per se* could contribute to the loss of E2-mediated vascular protection in postmenopausal women, in part by altering ERα expression and/or function.

Collectively, so far animal studies have helped to identify at least three factors that could explain the WHI controversy and reinforces the “timing hypothesis” on the vasculoprotective action of estrogens: 1) the duration of estrogenic deprivation, 2) vascular disease progression, and 3) aging of arteries *per se* (Figure 2 and Table 3). These three factors are intrinsically linked and may affect differently E2 action depending on the vascular function studied. In order to better understand effects of estrogens at menopause and during aging, it seems important to better delineate their mechanisms of action on the vascular wall.

## How Are Estrogen Receptor Expression and Function Impacted by Aging?

Estrogens rely on the fixation and activation of estrogen receptors to mediate their actions. To date, three estrogen receptors (ERs) have been identified, ERα, ERβ and, more recently, G protein-coupled estrogen receptor 1 (GPER) (Barton et al., 2018). GPER has been reported to mediate some beneficial vascular effects of estrogens such as protection against atherosclerosis and hypertension (Barton and Meyer, 2019) but these effects remained controversial due to conflicting results obtained with the different mouse models targeting this receptor (Zimmerman et al., 2016). More recently, identification of GPER selective agonists and antagonists such as G-1, G15, and G36, allowed to elucidate more specially its biological effects. In particular, use of G-1 *in vivo* demonstrated that activation of GPER induced endothelium-dependent vasodilation (Peixoto et al., 2017) and ameliorated experimental hypertension (Liu et al., 2016).

Both ERα and ERβ were reported to be expressed in human and animal vasculature, including in endothelial and smooth muscle cells (Mendelsohn and Karas, 1994). However, data on ERβ expression are still controversial. Indeed, despite the discovery of ERβ variants and isoforms, the majority of the studies were done with inadequately validated antibodies (Božović et al., 2021). More research is therefore needed to investigate the expression and the role of ERβ in normal arterial physiology, as well as in cardiovascular diseases. Depending on the tissue, ERα and ERβ have been described to exhibit overlapping or inhibitory actions (Matthews and Gustafsson, 2003). Nevertheless, until now, using ERα and ERβ deficient mice, ERα, but not ERβ, was shown to mediate most of the vascular effects of estrogens (Arnal et al., 2010).

**FIGURE 3:**
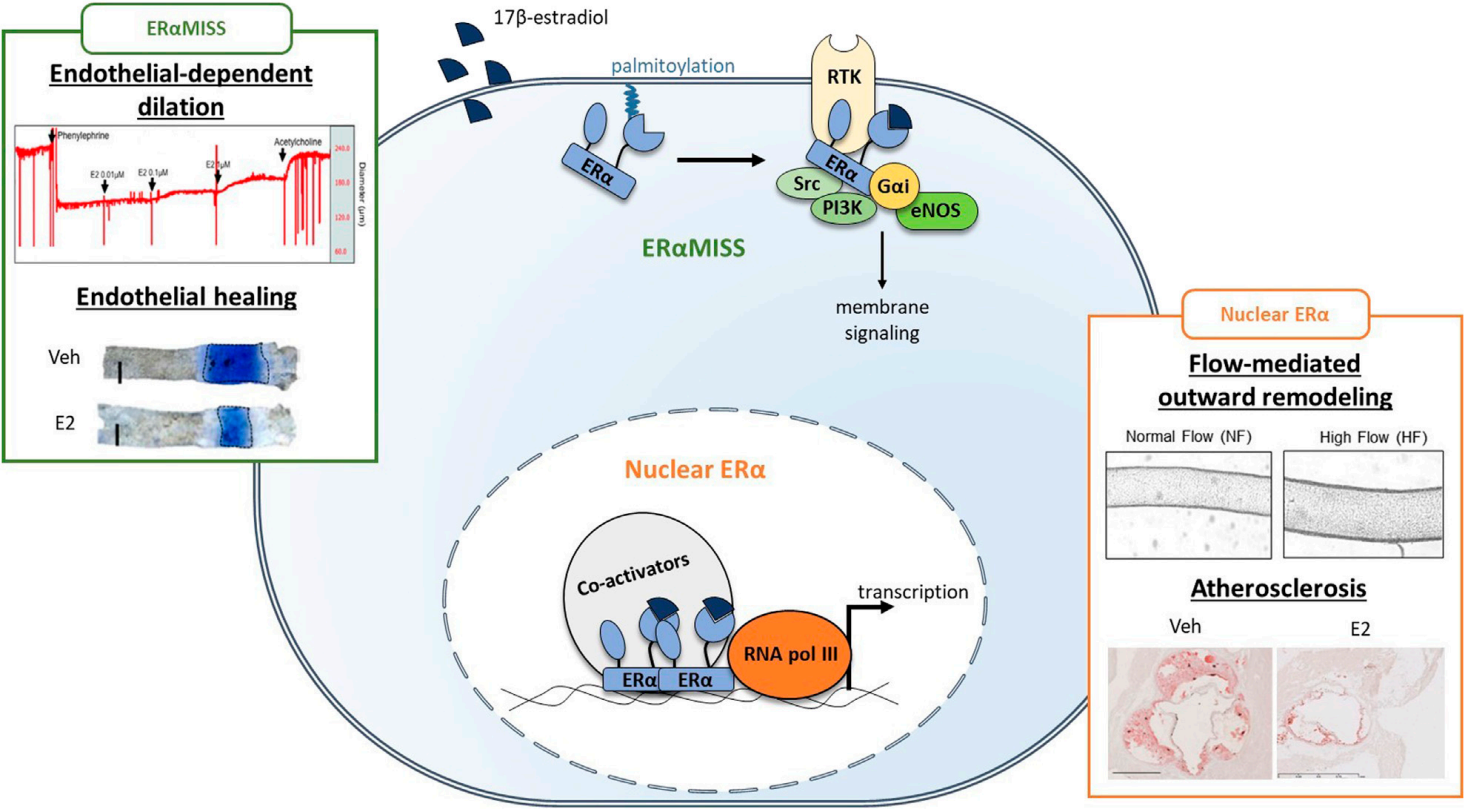
Respective role of ERαMISS and nuclear ERα in E2-mediated vascular protection in endothelial cells. Fixation of 17β-estradiol (E2) triggers conformational changes in ERα structure, leading to the recruitment of co-activators. Co-activators facilitate ERα binding to DNA and the recruitment of RNA polymerase (RNA pol III) which initiates transcription. In addition, ERα can localise to the plasma membrane after palmitoylation and mediates rapid membrane-initiated steroid signaling (ERαMISS) by interacting with different signaling pathway such as G protein, SRC, PI3K and eNOS. NO production and acceleration of endothelial healing in response to E2 depend on ERαMISS, while flow-mediated outward remodeling and prevention of atherosclerosis depend on nuclear ERα. Adapted from Guivarc’h et al. (2018), Adlanmerini et al. (2020), Zahreddine et al. (2020).

ERα belongs to the nuclear receptor family and acts classically as a transcription factor (Arnal et al., 2017; Hewitt and Korach, 2018). Briefly, fixation of E2 on its ligand binding domain (LBD) triggers conformational changes in ERα structure, which in turn lead to the recruitment of different co-activators on the activation function 1 and/or 2 (AF1 and AF2) leading to classical nuclear action (Figure 3). Co-activators possess histone acetyltransferase activity leading to chromatin opening and thus facilitate ERα binding to DNA through its DNA binding domain (DBD) and the recruitment of RNA polymerase which initiates RNA elongation. ERα can also regulate transcription indirectly after binding to other transcription factors such as AP-1 and SP1. In addition to its transcriptional activity, ERα also mediates rapid membrane-initiated steroid signaling (MISS). In particular, a part of ERα pool undergoes post-translational palmitoylation on cysteine-447 (in humans; cysteine-451 in mice) and localizes to caveolae where it interacts with various signaling pathway such as G protein, SRC, PI3K and eNOS (Arnal et al., 2017) (Figure 3). In addition to the “classic” full-length 66-kDa ERα (ERα66) which harbors both AF-1 and AF-2, two other isoforms of 46 kDa (ERα46) and 36 kDa (ERα36) have been characterized. ERα36 differs from ERα66 by lacking both transcriptional activation domains (AF-1 and AF-2) and encoding a unique 29 amino acid sequence (Wang et al., 2005). In contrast, ERα46 only lacks the first 173 N-terminal amino acids which harbors AF-1 and is thus completely identical to the amino acids 174 to 595 of ERα66. ERα46 has been reported to be expressed in endothelial cells and to be preferentially addressed at the plasma membrane (Li et al., 2003). The vascular role of the AF-1-deficient ERα46 isoform has been questioned *in vivo* using mice deficient in the ERα A/B domain (named ERaAF10), which express only a short 49-kDa isoform that is functionally similar to ERα46. These ERαAF-1^0^ mice revealed a complete infertility phenotype but preserved the three major vasculoprotective actions of E2 *i.e.* protection against atherosclerosis, increased of endothelial NO production and acceleration of reendothelialization (Billon-Galés et al., 2009b).

It is well established that serum E2 levels decrease with age in women, but how ERs and GPER expression in the arterial wall cell types is impacted by aging has been far less studied (Yang et al., 2021). Age-related diminution of ERs expression has been found in the retina of postmenopausal women (Ogueta et al., 1999) and the hypothalamus of middle-aged female rats (Chakraborty et al., 2003), however little is known about changes in ERs in the aging vasculature. This is of particular importance since gene dosage of ERs can impact greatly E2 mediated vascular response. Indeed, in ERα heterozygous female mice, a 2-fold decrease in ERα expression diminished E2 beneficial action on flow-mediated outward remodeling (Tarhouni et al., 2014), on endothelial healing (Brouchet et al., 2001), and on atheroprotection (Billon-Galés et al., 2009b). In the aorta of aged female SHRs (16 months) ERα and ERβ protein levels were slightly decreased compared to young animals (10 weeks), but this difference did not rich statistical significance (Wynne et al., 2004). In a mouse model of accelerated aging (senescence-accelerated prone mice), ERα mRNA levels were decreased while, unexpectedly, ERβ was increased (Novensà et al., 2011).

One mechanism that can explain the downregulation of gene expression is methylation of the CpG island in the gene promoter region which leads to permanent repression of transcription (Bird, 1986). Indeed, methylation of ERα promoter is associated with loss of ERα expression in human breast cancer cells (Ottaviano et al., 1994). Methylation of ERα gene can occur upon prolonged estrogenic deprivation in breast cancer cells and results in the incapacity for those cells to transcriptionally respond to E2 (Pink and Jordan, 1996). This could explain in part the “timing hypothesis” observed in late postmenopausal women, however effect of estrogen deprivation on the methylation status of ERs in vascular tissues has not been documented so far to our knowledge. Besides, accumulation of DNA methylation correlates positively with the age of human tissues and cell types and is considered as an “epigenetic clock” (Horvath, 2013). Age-related increase in ERα promoter methylation has been identified in the human cardiovascular system (Post et al., 1999). Methylation of ERα gene is more frequent in atherosclerotic plaques and correlates with the severity of the disease in patients (Huang et al., 2009). This is consistent with another study showing that ERα expression is decreased in atherosclerotic arteries of premenopausal women (Losordo et al., 1994). Thus, methylation of ERα with aging and vascular disease could contribute to the loss of E2 effects in elderly women. Another mechanism controlling ERα expression during aging could involve non-coding RNA transcripts, as a large number of them have been found to be crucial regulators of epigenetic modulation, transcription, and translation. To date, only few long non-coding RNAs and microRNAs have been identified as modulators of ERs expression, mainly in breast cancer cells (Vrtačnik et al., 2014; Wu et al., 2016). Non-coding RNAs associated with ER signaling may serve important regulatory roles in vascular aging and this field of investigation deserves to be deepened.

Globally, only a few studies have described changes in ERs expression in the vasculature during aging. More information is needed regarding ERs and GPER mRNA and protein levels in both middle-aged and aged animals but also in the different vascular beds. Importantly, E2 action can be mediated by ERα either in endothelial, smooth muscle and hematopoietic cells, in which expression of the receptor could vary differently.

### Role of ERα in Vascular Cell Subtypes and Aging

The crucial role of ERα in the endothelium was demonstrated using mouse models with tissue-specific deletion of ERα in endothelial cells with the Cre/Lox system under control of the Tie2 promoter. Indeed, the beneficial effects of E2 on the protection against atheroma (Billon-Galés et al., 2009a), NO production (Billon-Galés et al., 2009b), acceleration of endothelial healing (Toutain et al., 2009), and flow-mediated remodeling (Tarhouni et al., 2013) are all lost in Tie2Cre + ERα^lox/lox^ female mice. Endothelial ERα, which mediates the majority of E2 effects, is diminished by 33% in peripheral veins of postmenopausal women if compared to premenopausal women in their late follicular phase (high E2 levels) but not in their early follicular phase (low E2 levels) (Gavin et al., 2009). This result suggests that estrogen deprivation, rather than age, affects ERα expression. Influence of estrogen levels on the expression of ERα has also been described in female rats in which prolonged estrogenic deprivation led to decreased receptor expression in the aorta (Pinna et al., 2008) and the mesenteric artery (Tarhouni et al., 2014). However, estrogenic status is not the only factor influencing ERα expression, since a decrease of ERα expression in the aortic endothelium is observed in ovariectomized mouse model of accelerated senescence (Novensà et al., 2011).

The contribution of ERα in smooth muscle cells to E2 vascular action is more modest but still necessary for the prevention of neointima hyperplasia following mechanical injury of the mouse femoral artery which was demonstrated using the αSMACre^ERT2^ ERα^lox/lox^ mouse model leading to a specific deletion of ERα in smooth muscle cells (Smirnova et al., 2015). However, age-related changes in expression of ERα in smooth muscle cells remain to be better characterized, since one study described decrease in ERα protein levels in the aorta medial layer *in situ* in a model of accelerated aging (Novensà et al., 2011), whereas another found no difference in ERα levels in primary culture of aortic smooth muscle cells from young (10 weeks) and middle-aged (12 months) mice (Bowling et al., 2014). This later study should be considered cautiously as cell culture is known to elicit a repression of ERα expression.

Expression of ERα in hematopoietic cells also contributes to mediate E2 vascular protection. E2 effect on endothelial healing is lost in wild-type female mice grafted with ERα^−/−^ bone marrow. Thus, in addition to endothelial ERα, hematopoietic ERα is needed to mediate this action, at least in a context of post-irradiated young animals (Toutain et al., 2009). Atheroprotection by E2 could also depend on the expression of ERα in the immune compartment, since this effect is abrogated in RAG^−/−^/ApoE^−/−^ mice deficient in mature B and T lymphocytes (Elhage et al., 2000). Thus, how aging affects expression of ERα in hematopoietic cells should be also examined, as they contribute to some E2 vascular action. To date, only one study have described a decrease in ERα expression in bone-marrow derived macrophages from 12-month-old mice compared to 10-weeks-old mice (Bowling et al., 2014).

While endothelial ERα expression seems to be influenced by both estrogenic deprivation and aging, how smooth muscle and hematopoietic ERα are affected remains unanswered. In addition to receptor global expression, ERα subfunctions could also be affected differently by aging because they rely on different partners and signaling pathways. To answer this question, it is important to delineate the role of nuclear and membrane ERα action on vascular protection. Analyzing expression of the splicing variants of ERα in vascular aging would be also informative.

### How Are Membrane and Nuclear ERα Impacted by Aging?

Delineation of the contribution of membrane *versus* nuclear ERα to estrogens vascular action has been achieved *in vivo* thanks to complementary mouse models and pharmacological tools (Figure 3). Indeed, development of new pharmacological tools targeting membrane ERα only, such as estrogen-dendrimer conjugate (EDC) and pathway preferential estrogens (PaPEs), allowed to demonstrate the crucial role of membrane signalization for NO production and endothelial healing *in vivo* (Chambliss et al., 2010; Madak-Erdogan et al., 2016). These observations were further confirmed using ERα^C451A^ mice, lacking membrane ERα, in which effect of E2 on NO production and endothelial healing were abrogated (Adlanmerini et al., 2014). On the contrary, in a mouse model of nuclear ERα loss of function (ERαAF2^0^ mice), these beneficial actions of E2 were fully preserved (Billon-Galés et al., 2011). Recently, we also highlighted the importance of membrane ERα/Gαi interaction to mediate this E2 beneficial effect using a mouse model with a point mutation in ERα-Gαi interaction domain (ERα^R264A^ mice) (Adlanmerini et al., 2020). As endothelial-dependent relaxation to E2 is diminished in aged animal models, we could wonder if membrane ERα signaling is specifically lost with advance in age. Hence, this leads to two additional questions.

First, how ERα subcellular localisation is impacted by aging? This question cannot easily be answered since endogenous membrane ERα, which represents less than 5% of total ERα within the cell, is not observable using standard immunofluorescence approaches (Arnal et al., 2017). Nevertheless, some studies reported dysregulation of cysteine post-translational modifications, including palmitoylation, in old rodents, suggesting that ERα subcellular trafficking could be altered with age (Gu and Robinson, 2016).

The second question is could membrane ERα downstream signaling, *i.e.,* Gαi and eNOS, be impacted? On one hand, alterations in G-protein signaling during aging have been well established in the central nervous system and may be implicated in the establishment of neurodegenerative diseases such as Alzheimer’s disease and Parkinson’s disease (de Oliveira et al., 2019). However, if and how these alterations arise in the cardiovascular system is far less recognized. While one research group found a 40% reduction in Gαi subunits in the aorta of 24-month-old rats compared to 6-month-old controls (Johnson et al., 1995), another found no significant difference in the same conditions (Mader et al., 1996). On the other hand, reduced NO production is a well-recognized marker of endothelial dysfunction and aging, which could originate from decrease eNOS expression, decrease availability of NO precursor (L-arginine), decrease availability in eNOS cofactor (BH4), and/or increase in endogenous NO inhibitor ADMA (Novella et al., 2012). Globally, these alterations in eNOS activity could explain the decrease in NO production and endothelial-dependent relaxation observed with age following the activation of membrane ERα. Because acceleration of endothelial healing in response to E2 relies also on 1) membrane ERα, 2) Gαi-ERα interaction and 3) presence (but not activity) of eNOS, possible alteration of this process during aging should be examined.

Because selective activation of membrane ERα with EDC increases NO production and accelerates endothelial healing with minimal impact on the reproductive system, uncoupling membrane and nuclear ERα was initially proposed as a therapeutic option to confer cardiovascular protection without increasing cancer risk in menopausal women (Chambliss et al., 2010). However, the importance of nuclear/transcriptional action of ERα in mediating atheroprotection was demonstrated using a mouse model invalidated for the nuclear actions of ERα (ERαAF2^0^/LDLr^−/−^ mice). In this mouse model, the protective effect of E2 against early atheroma was completely abrogated at the aortic sinus (Billon-Galés et al., 2011), as well as the protective effect of endogenous estrogens after *en face* analysis of the thoracic and abdominal aortas (Guivarc’h et al., 2018). Conversely, the protective effect of E2 was fully preserved in ERα^C451A^/LDLr^−/−^ mice and treatment with EDC or PaPE did not confer atheroprotection (Guivarc’h et al., 2018). Flow-mediated outward remodeling of the mesenteric artery is totally abrogated in ERαAF2^0^ mice whereas ERα^C451A^ mice are fully responsive to the increase in flow and develop outward remodeling (Guivarc’h et al., 2018). Altogether, nuclear ERα appears to play a prominent role in the vasculoprotective action of E2. Interestingly, none of these vascular actions, *i.e.,* prevention of atherosclerosis and FMR, seems to be altered by aging *per se*, with regards to available literature. Nevertheless, alterations in DNA methylation, histone modifications, and chromatin remodeling are now well recognized as hallmarks of global aging (López-Otín et al., 2013), and more particularly, aging of the cardiovascular system (Zhang et al., 2018). How these changes could interfere with nuclear ERα transcriptional activity and vascular actions should be now studied.

Among key questions, there is a need to elucidate the role of ERα in altered mechanotransduction, which is known to accelerate vascular aging. GPER has been identified as a mechanoreceptor in fibroblasts controlling cell polarization and focal adhesion dynamics (Lachowski et al., 2020). A specific action of ERα depending on its subcellular localization may position this receptor as a key player in age-related mechanobiology. This point is highly relevant since there is a continuum from membrane to nuclear mechanotransduction in muscular cells. It can be anticipated that a decrease of the muscle ERα receptor in its nuclear localization may be involved in chromatin remodeling and in expression of smooth muscle cells differentiation genes during aging.

Altogether, understanding the impact of vascular aging on ERα expression, localization, and function is of interest in order to develop new therapeutic strategies efficient to preserve cardiovascular health in postmenopausal women (Figure 2).

## How Recent Insights in the Mechanisms of Action of ERα Could Contribute to Optimize Selective Modulation of ERα in Medicine?

In addition to CEE and E2, which are commonly used in MHT, several selective estrogen receptor modulators (SERMs) have been developed and are currently approved for clinical practice. At the contrary to CEE and E2, which are ERα agonists, SERMs can exert ERα agonist or antagonist activity depending on tissues. SERMs would exert beneficial ER agonist actions on bone, vasculature and lipid parameters, while they may reduce unwanted side effects (mainly breast cancer) *via* their ER antagonist action.

Tamoxifen, the prototypical SERM, has been developed in the 1970s for the prevention and treatment of breast cancer (Jordan, 2019). Indeed, fixation of tamoxifen on ERα-LBD induces a conformational change different than the natural ligand E2, switching the recruitment of co-activators on ERα-AF2 domain to the recruitment of co-repressors, and thereby blocking transcriptional activity. However, tamoxifen can also exert agonist effects by recruiting co-activators on ERα-AF1 domain in bone, uterine and vascular tissues. Recently, a large prospective study enrolling 10,005 (United Kingdom) and 22,027 (United States) postmenopausal women with breast cancer found a lower risk in tamoxifen users to develop coronary artery disease, including myocardial infarction, compared to non-users (Matthews et al., 2020). The atheroprotective action of tamoxifen has been well documented in experimental models such as monkeys (Williams et al., 1997), rabbits, and ApoE^−/−^ (Reckless et al., 1997) and LDLr^−/−^ (Fontaine et al., 2013) mice. Interestingly, in mice, tamoxifen-mediated atheroprotection required ERα-AF1, whereas this function was dispensable for the atheroprotective action of E2 (Fontaine et al., 2013). Thus, even if E2 and tamoxifen act through the same receptor, ERα, the subfunctions involved are different. Recently, we also demonstrated that tamoxifen, as E2, accelerates endothelial healing after injury of the carotid artery. Unexpectedly, and at the contrary to E2 which required activation of endothelial membrane ERα to mediate this action, tamoxifen relied on the activation of nuclear ERα in smooth muscle cells to accelerate endothelial healing (Zahreddine et al., 2020). Thus, tamoxifen can modulate differently ERα subfunctions but also acts in different vascular cell types, revisiting the concept of SERM. To what extent this selective modulation of ERα, and in particular of nuclear ERα, could confer an advantage for the primary prevention of cardiovascular diseases in postmenopausal women will require further examination.

Raloxifene, a second generation SERM, is commercialized for prevention and treatment of osteoporosis in postmenopausal women. Effect of raloxifene on coronary outcomes was evaluated in the Raloxifene Use for The Heart (RUTH) trial, enrolling 10,000 postmenopausal women (Collins et al., 2009). A significant decrease of coronary events was found in women <60 years of age assigned to raloxifene compared to placebo. However, raloxifene, just as tamoxifen, has no beneficial effect on climacteric symptoms, particularly hot flushes limiting their therapeutic applications as MHT (Arnott et al., 2014). Other SERMs have been reported to confer cardiovascular benefits (reviewed in Nandur et al., 2004). However, how these molecules modulate ERα to achieve their protective action and how aging affect their effect remains to be determined.

In addition to SERMs, estetrol (E4), a natural estrogen produced during pregnancy, has been authorized by the FDA for commercial distribution for contraception and is in phase III clinical trial for MHT. In mice, E4 was found to confer protection against atherosclerosis (Abot et al., 2014; Buscato et al., 2021), AngII-induced hypertension, and to promote FMR (Guivarc’h et al., 2018). Interestingly, E4 selectively modulates nuclear ERα (Abot et al., 2014) and was found associated to a unique transcriptional signature in female mice liver, different from E2 (Buscato et al., 2021), suggesting that this estrogen shares common properties with SERMs. Because nuclear ERα actions may be preserved during aging compared to membrane signaling (Guivarc’h et al., 2020), selective modulation by E4 could represent an attractive option for the prevention of CVD in postmenopausal women.

## Could Estrogen-ER Action Also Interfere With Vascular Aging in Men?

**FIGURE 4:**
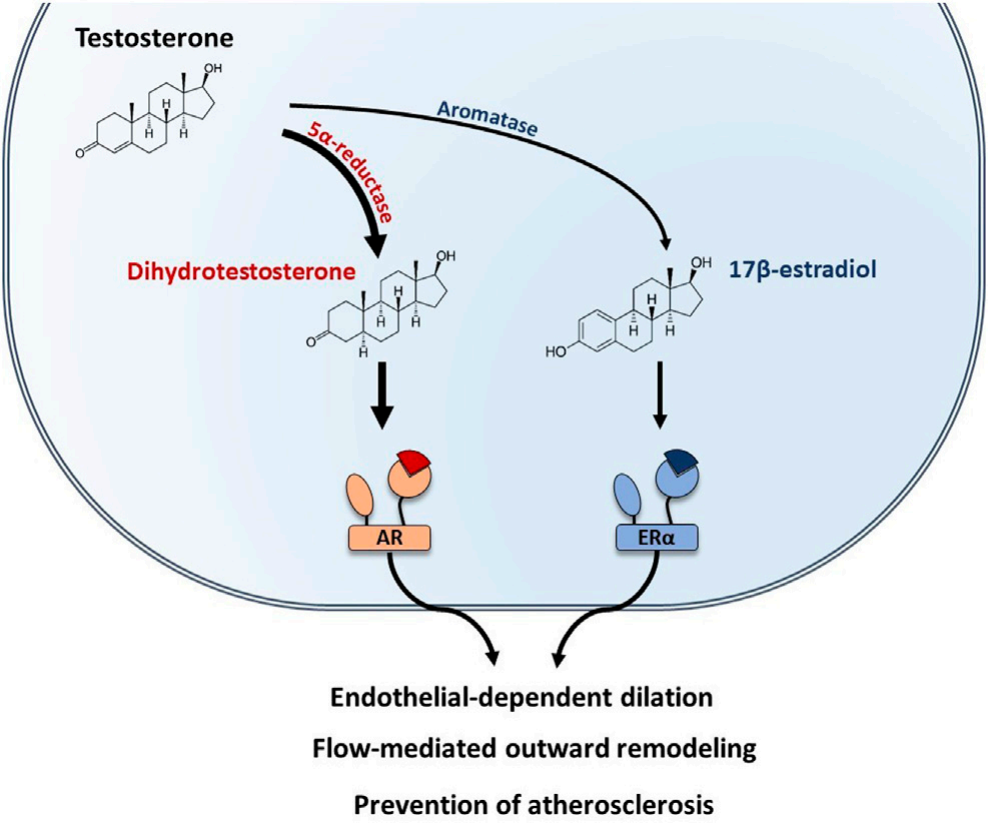
Testosterone-mediated vascular protection is mediated by both AR and ERα in males. In males, testosterone is the predominant sex hormone. Testosterone can be converted into dihydrotestosterone by the 5α-reductase and bind to the Androgen Receptor (AR). In addition, testosterone can also be converted into 17β-estradiol by the aromatase and act on ERα. Both AR and ERα mediate vascular protective effects in males, including endothelial-dependent dilation, protection against atherosclerosis, and flow-mediated remodeling.

In males, testosterone is the predominant sex hormone produced by the testis and its action is mediated by the Androgen Receptor (AR). AR-mediated vascular protective effects have been reviewed elsewhere and include endothelial-dependent dilation, protection against atherosclerosis (Boese et al., 2017), and flow-mediated remodeling (Chenu et al., 2017) (Figure 4). In addition, testosterone could be converted into E2 by local aromatization through aromatase activity in peripheral tissues. To better understand the role of E2-ERα actions in male, vasculature studies have combined the use of pharmacological tools (aromatase inhibitors, E2 administration) with transgenic mouse models deficient for either aromatase (ArKO) or ERα (ERαKO). E2-mediated endothelial-dependent dilation in males was demonstrated in the aorta of ArKO mice in which relaxation in response to testosterone was impaired (Kimura et al., 2003). This result was further supported by a clinical study in which young men treated with aromatase inhibitor for 6 weeks had a reduction in both E2 levels and FMD (Lew et al., 2003). Role of sex hormones in mediating FMR in males was demonstrated in orchidectomized mice in which remodeling in response to flow was lost in the mesenteric arteries, while E2 treatment of orchidectomized male mice restored FMR efficiently (Chenu et al., 2017). In addition, Nathan and coworkers demonstrated that E2 signaling also participates to the prevention of atherosclerosis in males, since the atheroprotective effect of testosterone was fully abrogated by aromatase inhibitor treatment of LDLr^−/−^ male mice (Nathan et al., 2001). Altogether, these results highlighted that conversion of testosterone to E2 is necessary to preserve endothelial function in males (Figure 4).

The importance of ERα in the cardiovascular system in men has been highlighted by the finding that mutation of *esr1* in a man was associated with impaired FMD and premature atherosclerosis (Sudhir et al., 1997b; 1997a). Moreover, polymorphisms in aromatase gene are associated with coronary artery disease and hypertension in men (Cooke et al., 2017)

While in women aging is associated to an abrupt decline in E2 levels, testosterone decrease in men is more gradual and highly variable between individuals. Several clinical studies have shown that low serum testosterone in middle-age and elderly men correlate with higher progression of atherosclerosis markers (van den Beld et al., 2003; Muller et al., 2004; Mäkinen et al., 2005), as well as impaired endothelial function (Akishita et al., 2007). Because E2 synthesis is dependent on testosterone bioavailability, E2 levels also modestly decrease with age in men (Vermeulen et al., 2002) (Figure 1). Thus, reestablishing normal sex hormone levels in men could improve cardiovascular health. Testosterone replacement therapy has been historically used to treat hypogonadism in elderly men, however results from epidemiological studies and clinical trials have provided conflicting results regarding possible cardiovascular adverse effects (Kharaba et al., 2020). Because of these results, in 2015 the food and drug administration (FDA) in the US expressed serious concerns regarding the safety of testosterone therapy (Nguyen et al., 2015; Lenfant and Arnal, 2017). Consequently, recent research has focused on the development of alternative treatments for the management of hypogonadism in aging men (reviewed in Krzastek and Smith, 2020). Interestingly, SERMs have been shown as efficient strategies to increase endogenous testosterone levels in men, by blocking negative feedback of E2 on the hypothalamus (Krzastek and Smith, 2020). Thus, SERMs could confer cardiovascular protection in aged men, indirectly by restoring normal testosterone levels, and directly through activation of ERα in the vascular wall. Clomiphene citrate has been the most studied SERM for the treatment of hypogonadism and was associated to minimal adverse effects (Wheeler et al., 2019), however data on long term cardiovascular outcomes are not available yet. Tamoxifen can also improve testosterone deficiency in men but is associated to more adverse effects, including gastrointestinal disorders and thromboembolic events, than clomiphene citrate (Krzastek and Smith, 2020). Nevertheless, by restoring normal testosterone levels, tamoxifen was found to increase FMD in men with pre-established coronary artery disease (Clarke et al., 2001), confirming its promising potential in the management of CVD in men. Further clinical investigations are needed to confirm these results.

## Conclusion

Study of gender difference have identified early that women in their reproductive years are protected against CVD compared to men, and present better endothelial function, decreased CVD risk factors and less atherosclerosis progression. In addition to aging, which is the principal contributing factor for CVD, a link between the drastic decrease in female sex hormones at menopause and loss of cardiovascular benefits in women has been clearly established in epidemiological and clinical studies. Use of experimental animal models allowed to identify E2-ERα as the main mediators of this cardiovascular protection, however it is important to note that most of these studies have been conducted in young animals. The most relevant animal model of accelerated aging in response to estrogens and receptor changes is still debated. Unexpectedly, clinical trials with MHT led only to modest beneficial effects regarding cardiovascular protection and was even associated to coronary adverse events in >70-year-old postmenopausal women. Three factors could explain these results: prolonged estrogenic deprivation, atherosclerosis progression, and advance in age. Indeed, these factors either diminish or abrogate E2 protective effects in animal studies, however underlying mechanisms are still poorly understood. Only few studies have evaluated changes in ERα expression in the different vascular cell types during aging and how membrane and/or nuclear functions are altered respectively by age will need further investigations. This is of particular importance in order to develop therapeutic strategies efficient to preserve cardiovascular health in both postmenopausal women and elderly men.

## Abbreviations

AF, activation function; CAC, coronary arterial calcification; CEE, conjugated equine estrogens; CVD, cardiovascular diseases; E2, 17β-estradiol; E4, estetrol; EDC, estrogen dendrimer conjugate; ER, estrogen receptor; FMD, flow-mediated dilation; FMR, flow-mediated outward remodeling; IMT, intima-media thickness; LBD, ligand binding domain; MHT, menopausal hormone therapy; MPA, medroxyprogesterone acetate; SERM, selective estrogen receptor modulator.
